# Development and validation of an early predictive model for hemiplegic shoulder pain: a comparative study of logistic regression, support vector machine, and random forest

**DOI:** 10.3389/fneur.2025.1612222

**Published:** 2025-06-18

**Authors:** Qiang Wu, Fang Zhang, Yuchang Fei, Zhenfen Sima, Shanshan Gong, Qifeng Tong, Qingchuan Jiao, Hao Wu, Jianqiu Gong

**Affiliations:** ^1^Department of Rehabilitation Medicine, The First Affiliated Hospital, Shaoxing University, Shaoxing, Zhejiang, China; ^2^School of Medicine, Shaoxing University, Shaoxing, Zhejiang, China; ^3^Department of Integrated Chinese and Western Medicine, The First People's Hospital of Jiashan, Jiaxing, Zhejiang, China; ^4^Department of Gastroenterology, The Third Affiliated Hospital of Zhejiang Chinese Medical University, Hangzhou, Zhejiang, China

**Keywords:** hemiplegic shoulder pain, prediction model, random forest, support vector machine, SHAP

## Abstract

**Objective:**

In this study, we aim to identify the predictive variables for hemiplegic shoulder pain (HSP) through machine learning algorithms, select the optimal model and predict the occurrence of HSP.

**Methods:**

Data of 332 stroke patients admitted to a tertiary hospital in Zhejiang Province from January 2022 to January 2023 were collected. After screening predictive variables by LASSO regression, three predictive models selected using the LazyPredict package, namely logistic regression (LR), support vector machine (SVM) and random forest (RF), were established respectively. The performance parameters (accuracy, precision, recall, and F1 score) of the models were calculated, the receiver operating characteristic curve (ROC) and the decision curve analysis (DCA) were plotted to compare the performance of the three models. An explainability analysis (SHAP) was conducted on the optimal model.

**Results:**

The RF model performed the best, with accuracy: 0.90, precision: 0.89, recall: 0.88, F1 score: 0.86, AUC-ROC: 0.94, and the range of the threshold probability in DCA: 7%−99%. Based on the SHAP analysis of the explainability of the RF model, the contribution degrees of the early HSP predictive variables from high to low are as follows: multiple injuries, shoulder joint flexion (p), biceps tendon effusion, sensory disorder, supraspinatus tendinopathy, subluxation, diabetes, and age.

**Conclusion:**

The RF prediction model has a good predictive effect on HSP and has good clinical explainability. It can provide objective references for the early warning and stratified management of HSP.

## 1 Background

Hemiplegic shoulder pain (HSP) is one of the most common complications after stroke, with an incidence rate as high as 30%−84% among hospitalized rehabilitation patients (1). HSP not only delays the recovery of upper limb motor function but is also closely related to psychological problems such as sleep disorders, anxiety, and depression, seriously affecting the rehabilitation process and quality of life of patients (2). The etiology of HSP is relatively complex, involving mechanical injury, neurogenic factors, metabolic abnormalities, iatrogenic injury, etc. (3, 4). Currently, clinical interventions mainly focus passively on pain management and rarely adopt proactive preventive strategies (5, 6).

In clinical practice, we have found that there is usually a time lag between the onset of hemiplegia and the occurrence of shoulder pain (7). If an effective predictive model can be established during this delay to identify high-risk individuals and implement intervention measures early, it will be of great significance for improving the incidence and prognosis of HSP.

In recent years, the application of machine learning technologies in the field of rehabilitation has experienced remarkable growth, particularly in areas such as stroke rehabilitation, where it focuses on predicting motor function recovery (8), developing personalized rehabilitation plans (9), and forecasting long-term functional outcomes (10). In spinal cord injury management, machine learning is utilized for predicting motor or bladder function recovery and stratifying complication risks (11). Additionally, in various neurological conditions, its applications include predicting consciousness recovery after traumatic brain injury (12), providing early warnings for epileptic seizures (13), and monitoring disease progression in neurodegenerative disorders such as Parkinson's disease (14). Although numerous studies have clarified the risk factors of HSP, such as impaired motor function, diabetes, spasticity, subluxation, and sensory disorders (2, 7, 15), most of them are limited to retrospective analysis or single-factor association studies and lack systematic modeling and prediction tool development.

This study is based on machine learning algorithms, systematically integrating clinical characteristics, biomechanical parameters, and ultrasound imaging results to construct an HSP prediction model. By comparing the performance of three models: logistic regression (LR), support vector machine (SVM), and random forest (RF), the optimal model is selected to improve the disease risk stratification ability and guide individualized rehabilitation plans, thereby reducing the incidence of HSP and optimizing the allocation of medical resources.

## 2 Materials and methods

### 2.1 Study design and subjects

**Figure 1:**
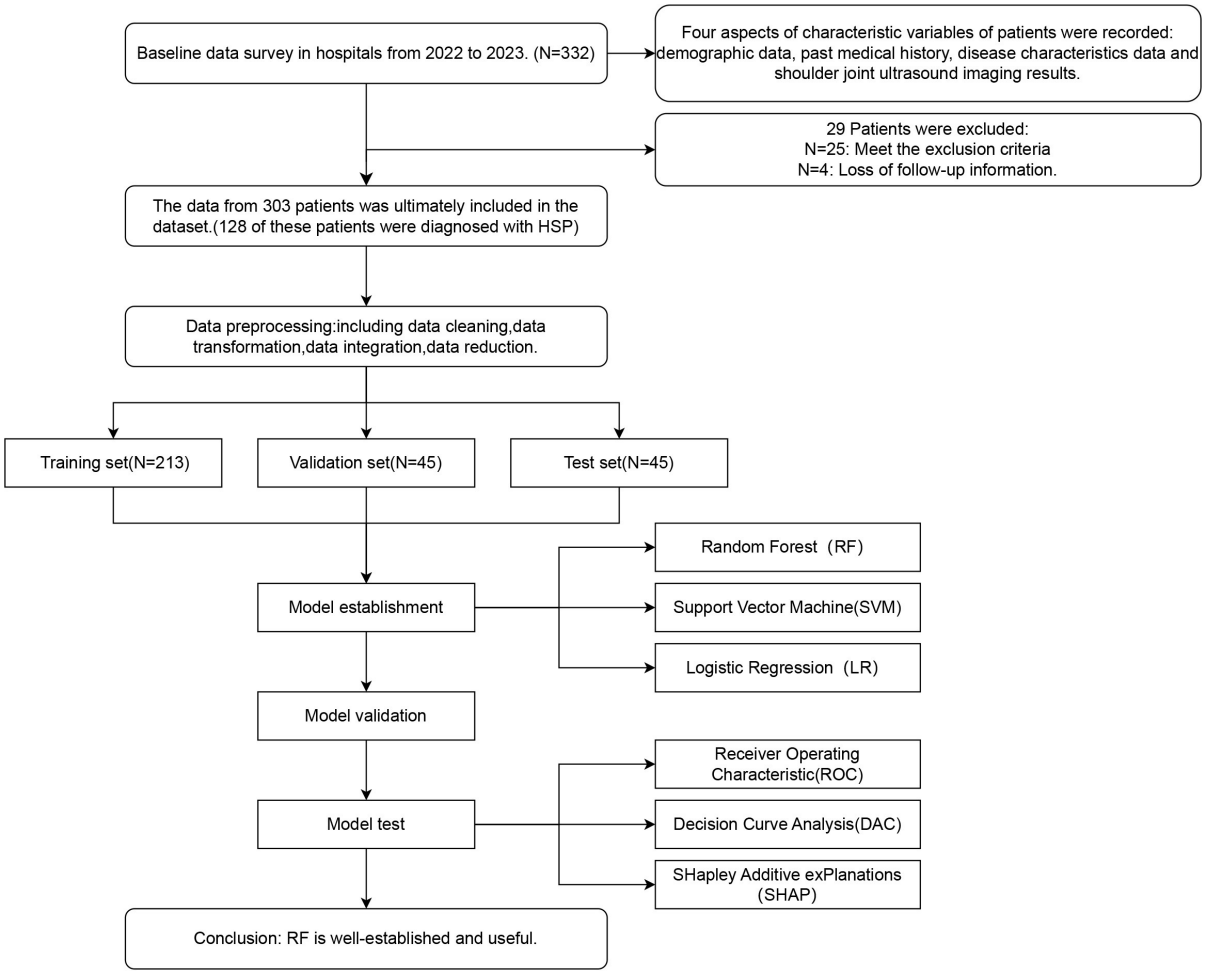
Research design flowchart.

We collected candidate variables from 332 inpatients at the Rehabilitation Medicine Department of a tertiary hospital in Zhejiang Province, China, from January 2022 to January 2023. After data cleaning and predictor screening, we developed three models using the candidate features. Model selection was based on both parameter performance and clinical decision utility curves, followed by SHAP-based explainability analysis of the optimal model (Study design workflow showed in Figure 1).

### 2.2 Study population

#### 2.2.1 Inclusion criteria

1) Compliance with the “Diagnostic Criteria for Cerebrovascular Diseases” established at the 4th National Conference on Cerebrovascular Diseases (1995).
2) Stroke diagnosis confirmed via Head CT or MRI.
3) Unilateral symptom onset with a disease duration ≤ 1 month.
4) Stable vital signs, ability to cooperate with assessments/examinations, and absence of aphasia or emotional disturbances.
5) Provision of informed consent and signed documentation.

#### 2.2.2 Diagnostic criteria for shoulder pain in hemiplegic patients

Presence of pain in the affected shoulder or upper limb within 48 h, including nocturnal pain, resting pain, and/or pain during passive movement of the affected limb, with a visual analog scale score ≥4.

#### 2.2.3 Exclusion criteria

1) Pre-existing brain injury or neurological disorders (e.g., brain tumors, spinal cord injury, and Parkinson's disease).
2) History of shoulder pain on the hemiplegic side prior to stroke.
3) Unstable vital signs or severe cognitive impairment.

#### 2.2.4 Data collection

A total of 332 patients were recruited. Following exclusions (*n* = 25) and dropouts due to missing follow-up data (*n* = 4), the final analysis included 303 patients.

### 2.3 Candidate variables

Candidate variables encompass four key aspects of patient characteristics: demographic data, past medical history, disease characteristics data, and shoulder joint ultrasound imaging results. Demographics include age and gender. Past medical history covers hypertension (HTN) and diabetes mellitus (DM). Disease characteristics consist of stroke type (cerebral infarction or intracerebral hemorrhage), hemiplegic side, Brunnstrom Stage (assessing upper limb motor function), Fugl–Meyer Assessment Score (FMA) for upper and hand function [FMA(U&H), comprising FMA(U), and FMA(H)], activities of daily living (ADL) via the Barthel Index, sensory disturbance (abnormal sensory or proprioceptive functions in the affected upper limb), passive shoulder flexion (maximum angle of passive flexion measured without pain using a joint angle gauge in a standard sitting position), spasticity (diagnosed when Modified Ashworth Scale ≥1), and subluxation (diagnosed when the distance between the glenoid process and humeral head exceeds one finger in a sitting position with naturally hanging upper limbs). Ultrasound imaging results include bicipital tendon effusion (bicipital TE, identified as a moon-shaped anechoic area around the long axis of the biceps long head, compressible and movable, with aggregation at the tendon in long-axis sections), Subacromial-subdeltoid bursa effusion (diagnosed when bursa effusion thickness >2 mm), joint effusion (diagnosed when the distance between the infraspinatus muscle and the posterior labrum of the glenoid >2 mm), supraspinatus tendinopathy (including tears, tendinitis, degeneration, calcification, etc.), subscapularis tendinopathy (including tears, tendinitis, degeneration, calcification, etc.), and multiple injuries (description of more than two lesion sites in the affected shoulder's soft tissue structure, such as the rotator cuff, acromion, acromion-trochanteric bursa, long head of the biceps tendon, glenohumeral joint, coracoid process, and greater tuberosity of the humerus).

### 2.4 Data analysis

The statistical analysis was performed using Jupyter Notebook (version 7.2.2) with Python to investigate differences in variables between the HSP group and non-HSP group, aiming to identify potential predictive factors. For continuous variables, data conforming to normal distribution (Shapiro–Wilk test, *P* > 0.05) were presented as mean ± standard deviation and compared using independent *t*-tests to evaluate group differences in means. Non-normally distributed data (Shapiro–Wilk test, *P* ≤ 0.05) were expressed as median (P25, P75) and analyzed using the Mann–Whitney *U* rank sum test to assess whether the groups originated from identical continuous distributions. Categorical variables were described as frequency percentages (%) and analyzed using the chi-square test to compare proportional differences between groups. The significance level was set at α = 0.05 for all analyses.

### 2.5 Dataset division

The dataset of 303 patients will be randomly divided into training, validation, and test sets at a ratio of 7:1.5:1.5 during the modeling process. The training set is used for model training, the validation set serves for hyperparameter optimization and model selection, while the entirely independent test set is reserved exclusively for final evaluation. This allocation ultimately results in 213 samples in the training set, 45 samples in the validation set, and 45 samples in the test set.

### 2.6 Model construction and comparison

We used Jupyter Notebook (v7.2.2) with Python to develop, validate, and compare predictive models. Only the variables in the training set underwent *z*-score standardization before analysis. Initial high-correlation variables were clinically pre-screened using Matplotlib-generated heatmaps, followed by multicollinearity assessment. Subsequently, the LASSO regression method from the scikit-learn was employed to screen important predictive factors. This method achieves this by incorporating L1 regularization into the loss function of traditional regression. When the regularization strength λ is sufficiently large, the coefficients of insignificant features are forced to zero, effectively automating the feature selection process. This simplifies the model and enhances its robustness.

We utilized a benchmarking approach (via the LazyPredict package) to rapidly compare the performance of 24 algorithms, and selected the top six models (representing the top 25% ranked by AUC scores) for further development and evaluation (16). These models were analyzed from the perspectives of metric performance and clinical interpretability to identify three representative models for subsequent refinement and assessment.

We refined LR, SVM, and RF models using scikit-learn. Hyperparameters were optimized via the scikit-learn class GridSearchCV with repeated 10-fold cross-validation (10 repeats) conducted on the training set. This procedure identified the optimal parameter combinations (e.g., number of trees and maximum depth for RF). The model's performance was evaluated on a test set using metrics such as accuracy, precision, recall, F1 score, and ROC-AUC. Accuracy measures the overall correctness of the model's predictions, that is, the proportion of correctly predicted samples out of the total samples. Precision focuses on the proportion of true positives among the samples predicted as positive, reflecting the accuracy of the predictions. Recall measures the proportion of actual positive samples that were correctly predicted as positive, indicating the model's ability to identify positive instances. The F1 score, as the harmonic mean of precision and recall, provides a comprehensive evaluation of the model's performance. ROC-AUC, represented by the area under the ROC curve, assesses the model's ability to distinguish between positive and negative instances (17). Decision curve analysis (dcapy library) further assessed clinical utility by visualizing net benefits across decision thresholds. The core principle of DCA lies in comparing the net benefits of different models against two extreme strategies (assuming all patients receive interventions or none receive interventions). By quantifying the differences in net benefits across varying clinical decision thresholds, DCA assesses the practical value of the models under diverse risk tolerance scenarios (18). Finally, SHAP summary plots were generated to interpret feature contributions to predictions.

## 3 Result

### 3.1 Baseline of clinical data

In our study, 303 individuals who met the inclusion criteria were enrolled. This included 79 male patients with hereditary spastic paraplegia (HSP) aged 45–87 years and 49 female HSP patients aged 43–84 years, as well as 101 male non-HSP patients aged 46–87 years and 74 female non-HSP patients aged 45–88 years. All subjects underwent standardized clinical evaluations and shoulder joint ultrasonography at enrollment. Clinical characteristics are fully detailed in Table 1.

**Table 1:** Baseline characteristics of the study cohort.

**Characteristics**	**Classify**	**HSP (*n* = 128)**	**non-HSP (*n* = 175)**	***P*-value**
Age		64.91 ± 9.97	65.55 ± 10.50	0.59
Sex	Male	79 (61.7%)	101 (57.7%)	0.46
	Female	49 (38.3%)	74 (42.3%)	
Type	CI	80 (62.5%)	123 (70.3%)	0.16
	ICH	48 (37.5%)	52 (29.7%)	
DM	Yes	111 (86.7%)	122 (69.7%)	<0.001
HTN	Yes	104 (81.3%)	131 (74.9%)	0.22
Side	L	68 (53.1%)	94 (53.7%)	0.92
	R	60 (46.9%)	81 (46.3%)	
Brunnstrom(U)		2.22 ± 0.98	2.67 ± 1.09	<0.001
Brunnstrom (H)		1.86 ± 1.13	2.18 ± 1.26	0.02
FMA(U)		8.86 ± 6.94	11.33 ± 8.45	0.01
FMA(H)		2.64 ± 4.30	3.84 ± 5.05	0.03
FMA (U&H)		11.50 ± 10.53	15.17 ± 12.56	0.01
ADL		25.73 ± 14.03	29.68 ± 18.20	0.03
Sensory disturbance	Yes	72 (56.3%)	46 (26.3%)	<0.001
Shoulder flexion (p)		118.03 ± 30.16	147.65 ± 22.53	<0.001
Spasticity	Yes	51 (39.8%)	28 (16.0%)	<0.001
Subluxation	Yes	52 (40.6%)	19 (10.9%)	<0.001
Bicipital TE (mm)		2.42 ± 1.66	1.07 ± 1.38	<0.001
SSB effusion (mm)		2.31 ± 2.45	0.40 ± 1.27	<0.001
Joint effusion (mm)		1.81 ± 4.94	0.74 ± 3.07	0.03
SS tendinopathy		0.41 ± 0.49	0.07 ± 0.26	<0.001
ST tendinopathy		0.05 ± 0.23	0.04 ± 0.20	0.56
Multiple injuries		0.90 ± 0.30	0.16 ± 0.37	<0.001

CI, cerebral infarction; ICH, intracerebral hemorrhage; DM, diabetes mellitus; HTN, hypertension; FMA, Fugl–Meyer Assessment Score; U, upper extremity; H, hand; TE, tendon effusion; SSB, subacromial-subdeltoid bursa; SS, supraspinatus; ST, subscapularis.

### 3.2 Screening of characteristic variables

**Figure 2:**
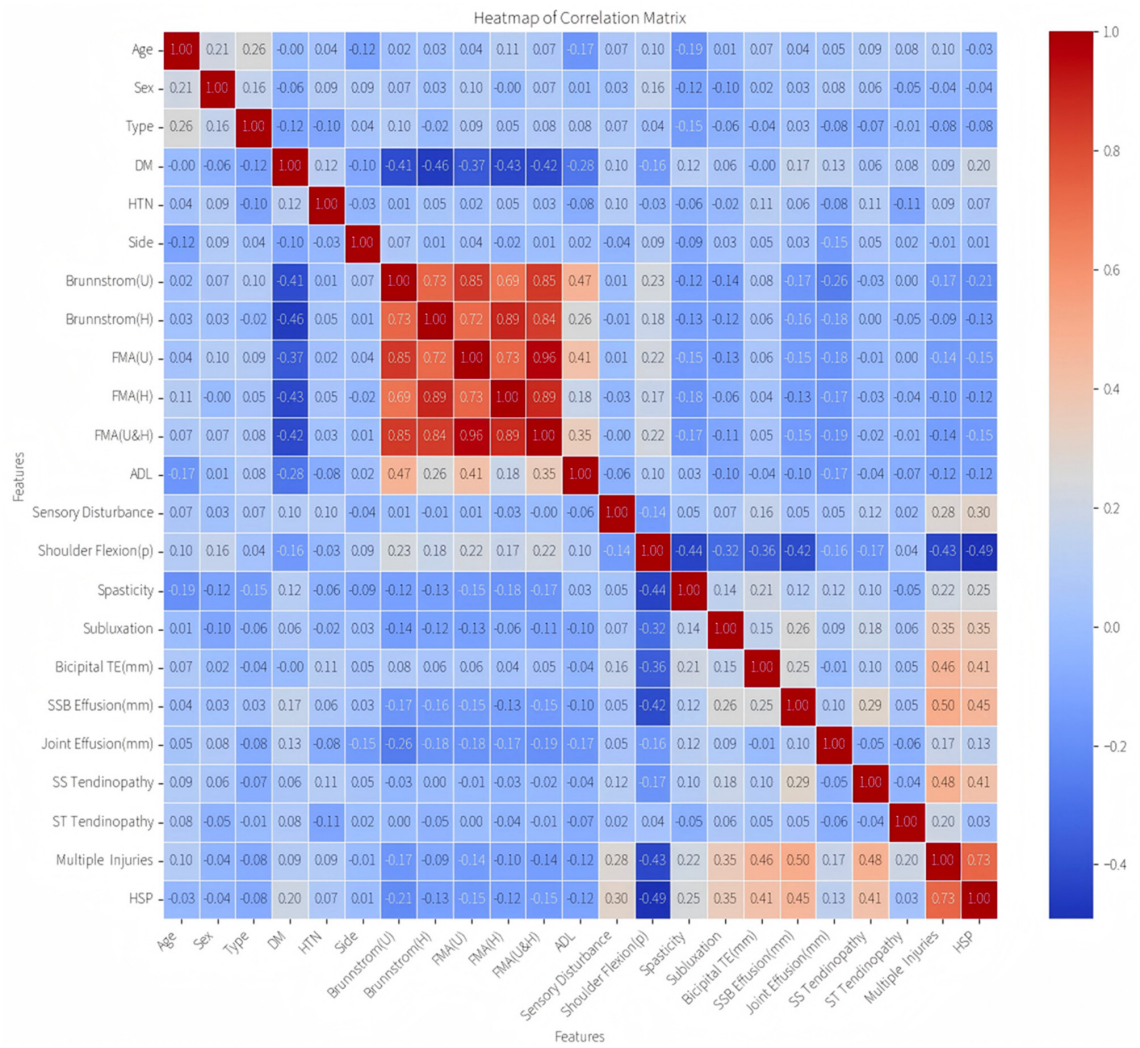
Correlation heatmap of characteristic variables.

Spearman's rank correlation analysis revealed a cluster of highly correlated variables (Figure 2): Brunnstrom stages (U/H) and FMA scores (U/H/U&H), reflecting inherent construct overlaps in motor impairment evaluation. To mitigate multicollinearity, four redundant variables were excluded, retaining Brunnstrom stages (U) as the representative metric based on its maximal correlation magnitude and clinical relevance.

**Figure 3:**
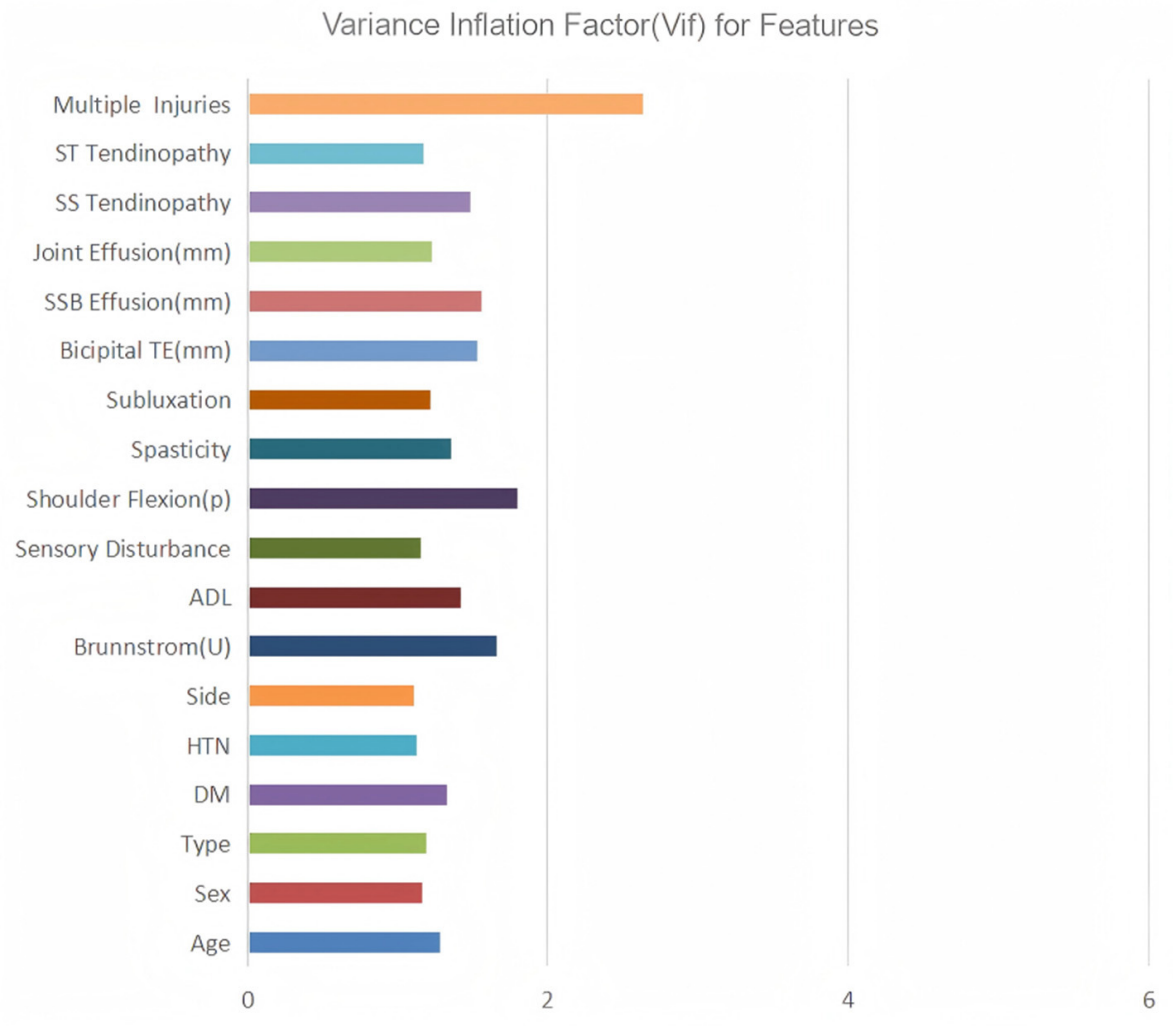
Variance inflation factor (VIF) for features.

**Figure 4:**
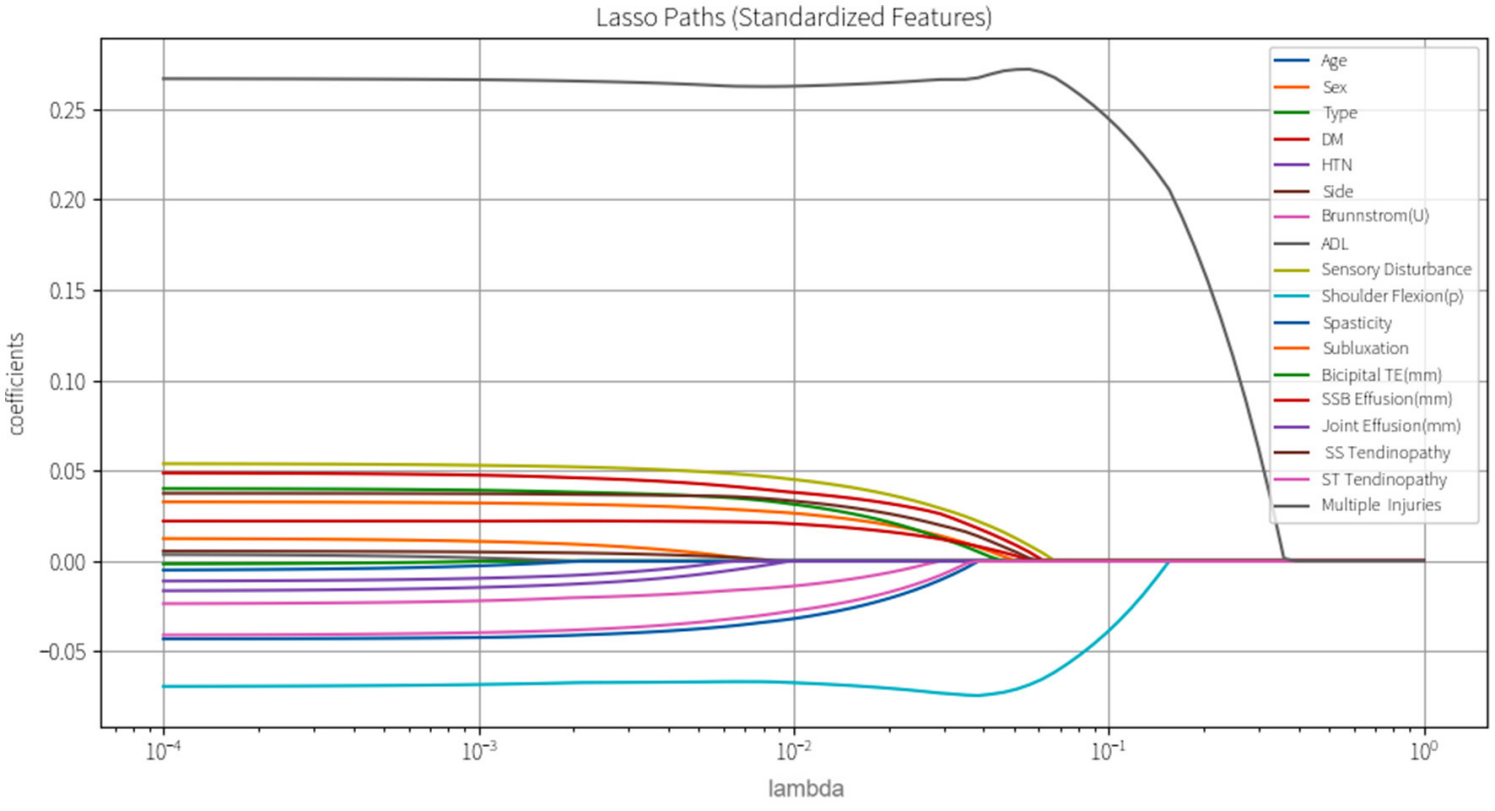
LASSO coefficient profile plot for feature variables.

**Figure 5:**
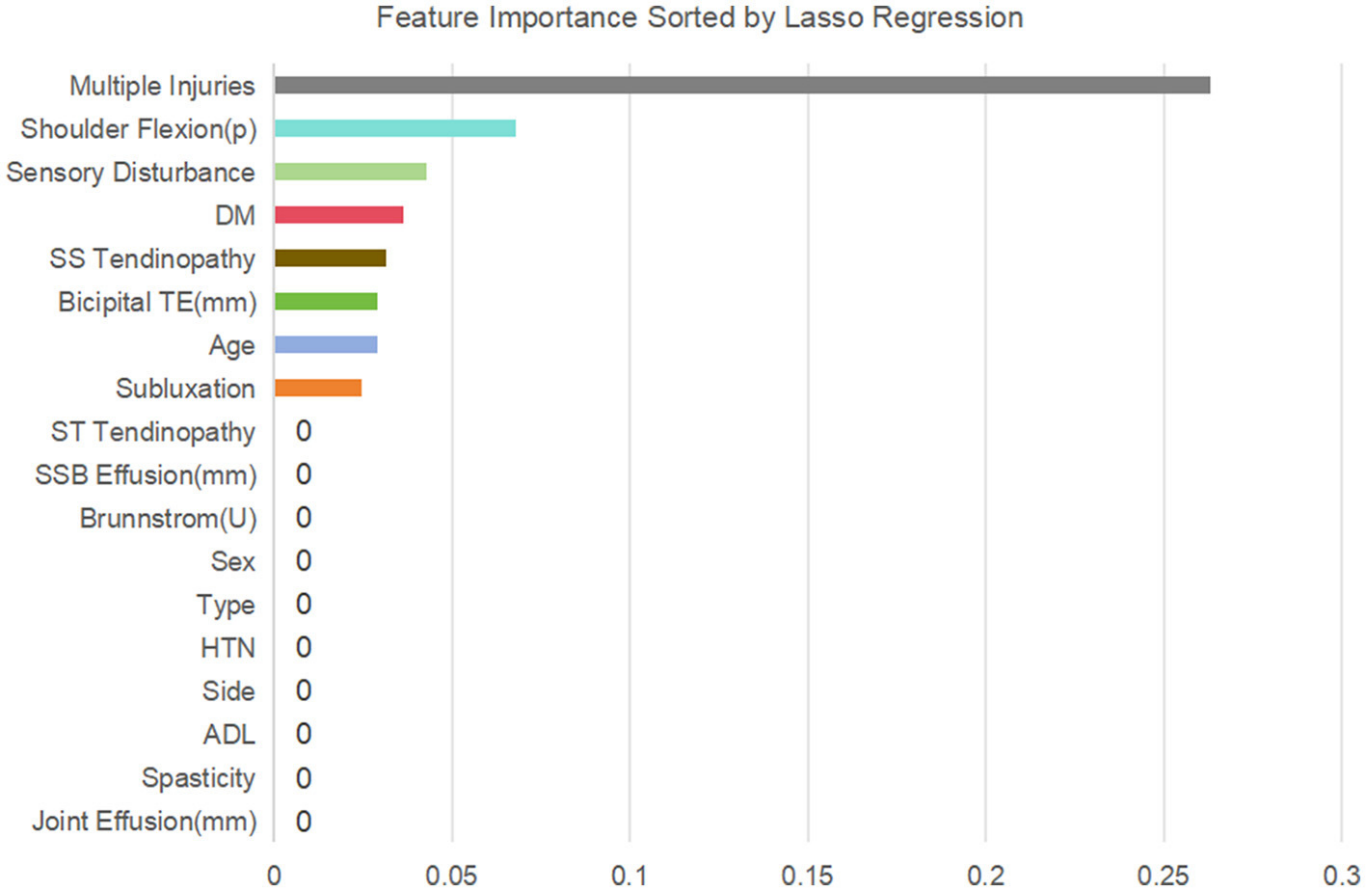
Feature importance sorted by lasso regression.

Subsequently, variance inflation factor (VIF) analysis was conducted to assess multicollinearity (Figure 3). The results showed that the VIF of the remaining 18 variables was all < 10, indicating that their collinearity level was acceptable and suitable for inclusion in the subsequent analysis (19). Considering the linear variables among these variables, in order to further optimize the model complexity and prevent overfitting, 10-fold cross-validation LASSO regression was implemented (20). The optimal penalty coefficient λ was determined to be 0.041 (Figure 4), and we finally obtained eight variables (Figure 5), from high to low: multiple injuries, shoulder flexion (p), sensory disturbance, DM, SS tendinopathy, bicipital TE, age and subluxation.

### 3.3 Model selection, establishment, and evaluation

We selected three representative models for further optimization and evaluation using the LazyPredict package, considering both rapid performance benchmarking and clinical interpretability (Table 2): LR model was chosen as the most interpretable representative, SVM model as the highest-performing option, and RF model as a balanced choice combining clinical interpretability with robust performance.

**Table 2:** Benchmarking for initial screening and comparison of models.

**Model**	**AUC**	**Accuracy**	**F1 score**	**Specificity**	**Training time (s)**	Explainability^*^
SVM	0.885	0.834	0.812	0.852	23.5	C
Random forest	0.868	0.821	0.809	0.837	18.7	B
XGBoost	0.829	0.829	0.801	0.841	12.4	C
LR	0.827	0.818	0.803	0.843	1.2	A
LightGBM	0.831	0.832	0.807	0.846	8.9	C
Naive Bayes	0.810	0.798	0.790	0.825	3.4	A

* Provide an explainability rating by integrating model prediction principles.

A, Excellent; B, Good; and C, Fair.

**Figure 6:**
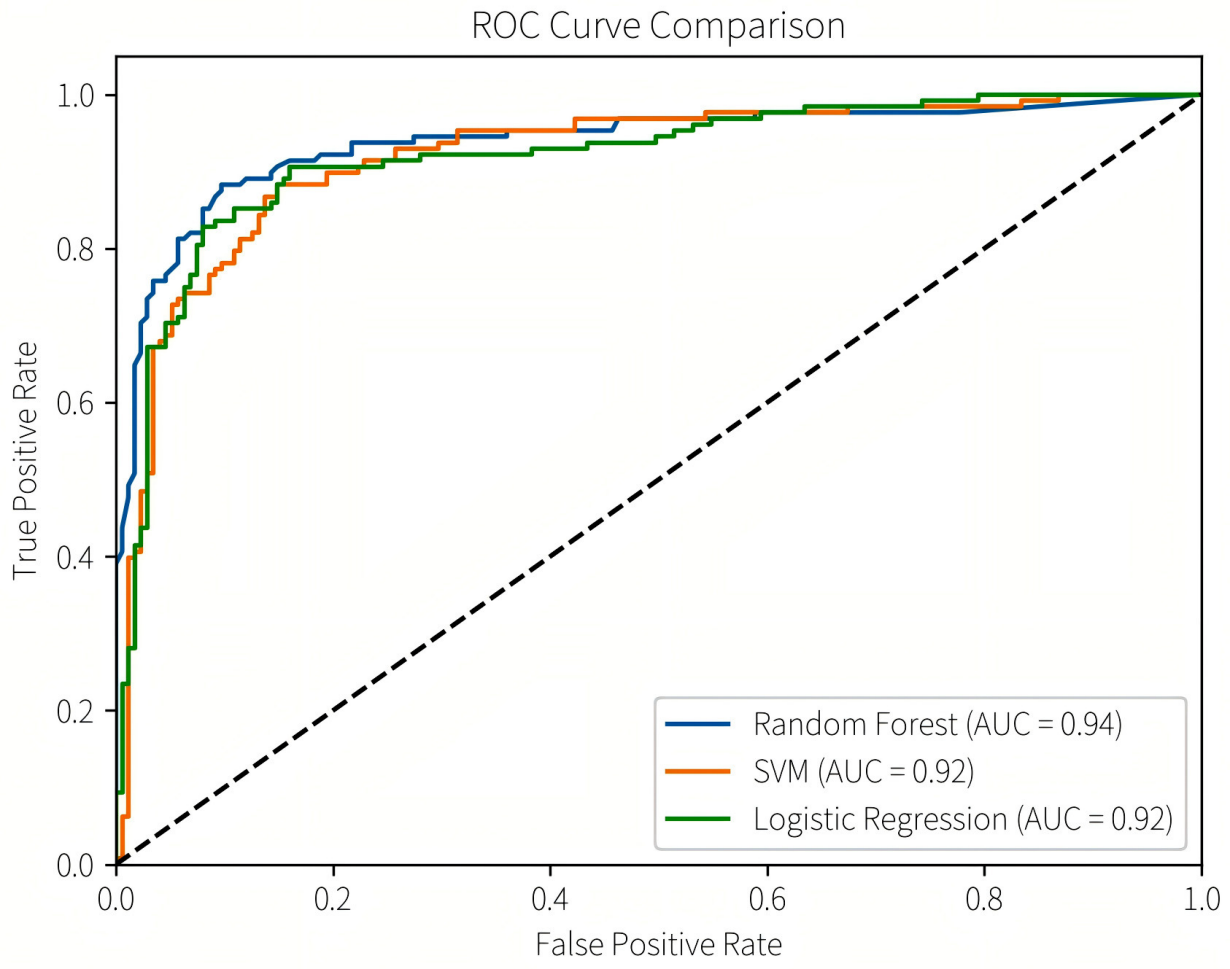
ROC curve comparison.

Subsequently, we conducted model refinement and evaluation on these three selected models. The performance parameters of the three models are compared as follows: the AUC-ROC values of the three models are all >0.8 (Figure 6), indicating that they all have good predictive performance. Based on the Delong test, the AUC-ROC value of the RF model is superior to that of the SVM model and the LR model, and the differences are statistically significant (*P* < 0.05). There is no statistically significant difference in the AUC values between the SVM model and the LR model.

**Figure 7:**
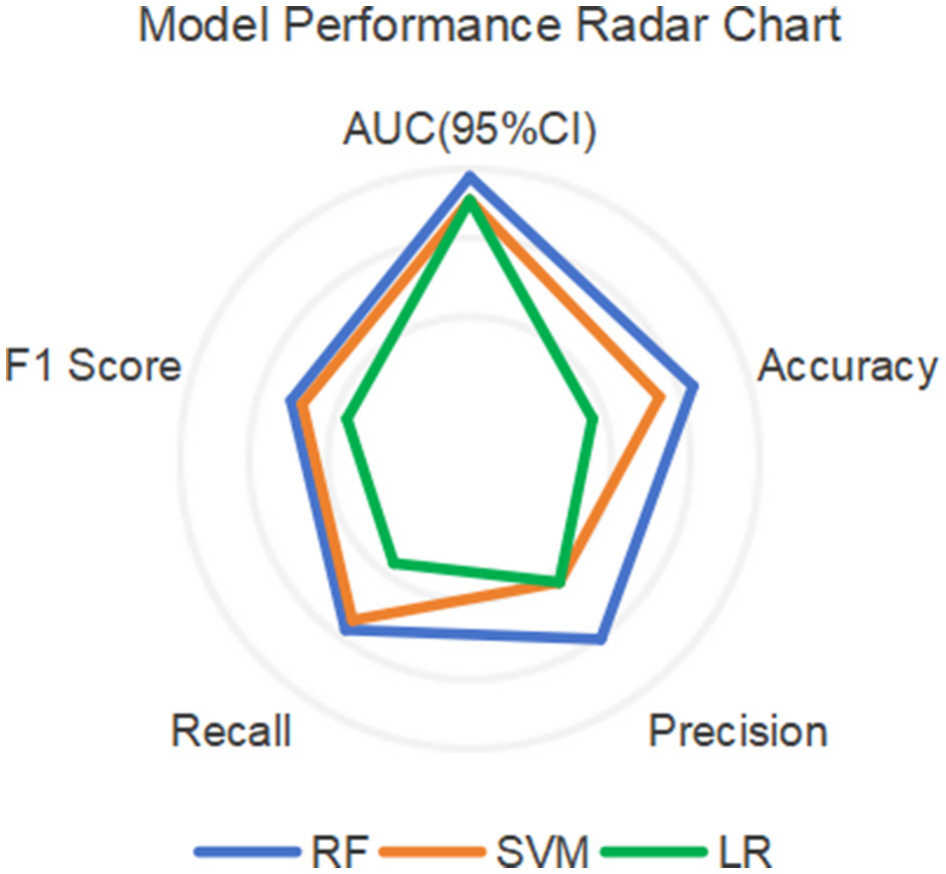
Radar chart of model performance.

Among other performance parameters, the models with the highest accuracy, precision, recall rate and F1 score are all RF models (Table 3). In the radar chart of core performance metrics (Figure 7), an evaluation framework is established using five key dimensions, where the area enclosed by the score contours of each model directly reflects their comprehensive performance. The comparative results demonstrate that both the random forest (RF) and support vector machine (SVM) models significantly outperform the traditional logistic regression (LR) model, underscoring the superior performance of machine learning algorithms over classical statistical models in complex problem modeling scenarios.

**Table 3:** Model performance comparison.

**Model**	**Optimized hyperparameters**	**AUC (95%CI)**	**Accuracy**	**Precision**	**Recall**	**F1 score**	***P* value**
RF	C = 10	0.94	0.90	0.89	0.88	0.86	0.034^*^
SVM	C = 100, gamma = 0.001	0.92	0.87	0.83	0.87	0.85	0.823
LR	n_estimators = 100, max_depth = 10	0.92	0.81	0.83	0.81	0.81	

* Compared with the LR model, *P* < 0.05.

RF, random forest; SVM, support vector machine; LR, logistic regression.

**Figure 8:**
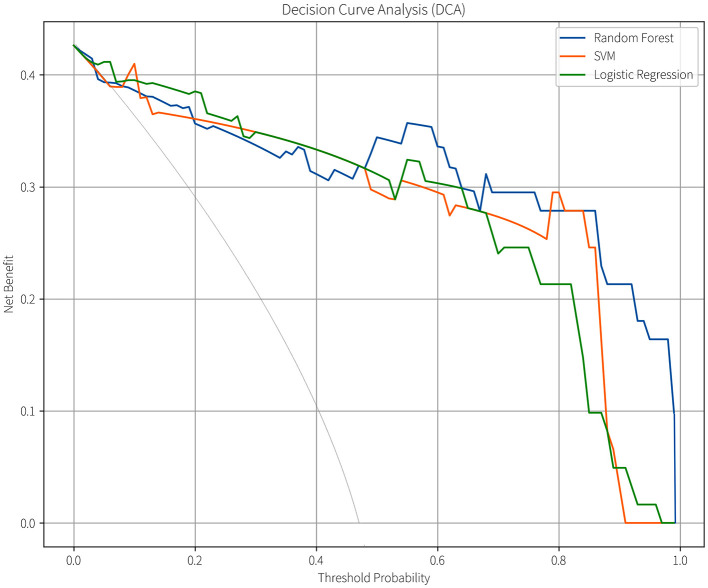
Comparison of DCA curves for the models.

Given that all three predictive models output continuous risk probability values and the decision-making value of probabilistic risk assessment is increasingly prominent in clinical machine learning applications (21), we further compared their clinical utility using decision curve analysis (DCA; Figure 8). In this figure, the horizontal axis represents the threshold probability, defined as the minimum probability threshold for initiating an intervention in clinical decision-making. The vertical axis denotes the model's net benefit value. When the model's net benefit curve exceeds the baseline curves of “treat all” and “treat none” strategies within a specific threshold range, it demonstrates that the model possesses clinical utility and practical value within that threshold interval. Additionally, a broader range of beneficial threshold probabilities can accommodate clinical decision-making preferences spanning from permissive (low threshold probabilities) to stringent (high threshold probabilities), enabling applicability across diverse risk stratification scenarios (18). All three models demonstrated clinical value within specific threshold probability ranges, the effective range of the SVM model was the narrowest (8%−89%), while that of the LR model was broader (5%−96%). The RF model had the widest range of clinical practicability across different thresholds (7%−99%), indicating its stronger adaptability in various clinical scenarios.

### 3.4 Explainability analysis

**Figure 9:**
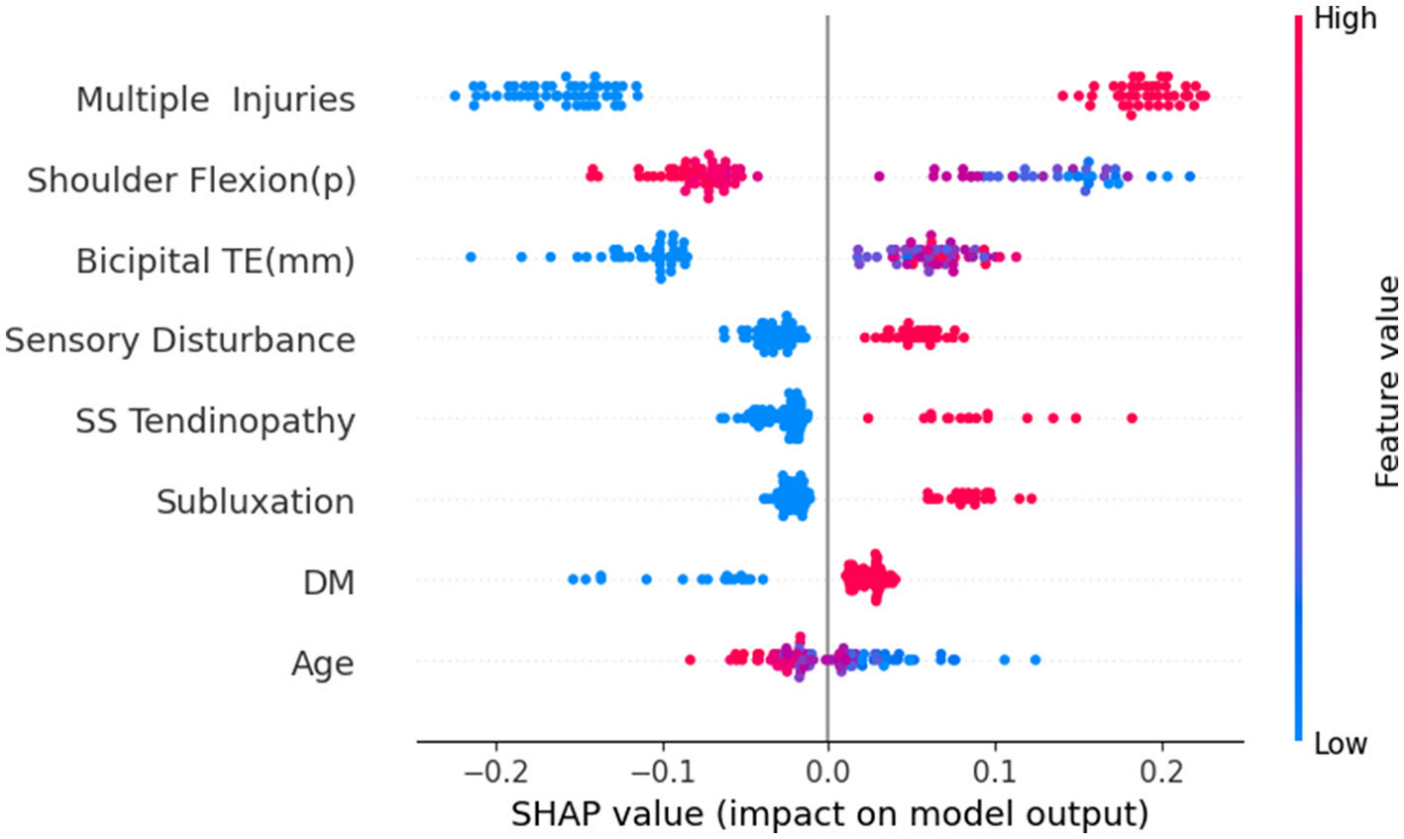
SHAP summary plot for the RF model.

Through the testing of the model and the analysis of its clinical practicability, we finally chose the RF model as the optimal model for predicting the risk of HSP. Subsequently, we conducted SHAP analysis on the RF model to analyze its explainability. Figure 9 is a summary chart of SHAP explanations for eight feature variables. Each point in the chart represents a sample. The *x*-axis represents SHAP values, where the left side of the *y*-axis indicates negative contributions and the right side denotes positive contributions, while the *y*-axis lists the names of feature variables. Red dots represent variables with larger SHAP values, and the darker the color, the greater the impact on the model output. Blue dots represent variables with smaller SHAP values, and the darker the color, the smaller the impact on the model output.

We found that multiple injuries has the widest distribution, indicating that this variable has the greatest impact on the output results of the RF model. Moreover, the red dots are located on the right side of the vertical axis, suggesting that it is proportional to the SHAP value. This indicates that patients with multiple injuries on the shoulder have a higher risk of developing HSP. Similarly, conditions like Bicipital TE, sensory disturbance, SS tendinopathy, subluxation, and DM all positively influence the risk of developing HSP. Red dots predominantly on the left side of the vertical axis for shoulder flexion (p) and age indicate that patients with greater shoulder flexion (p) or older age tend to have a lower risk of HSP.

**Figure 10:**
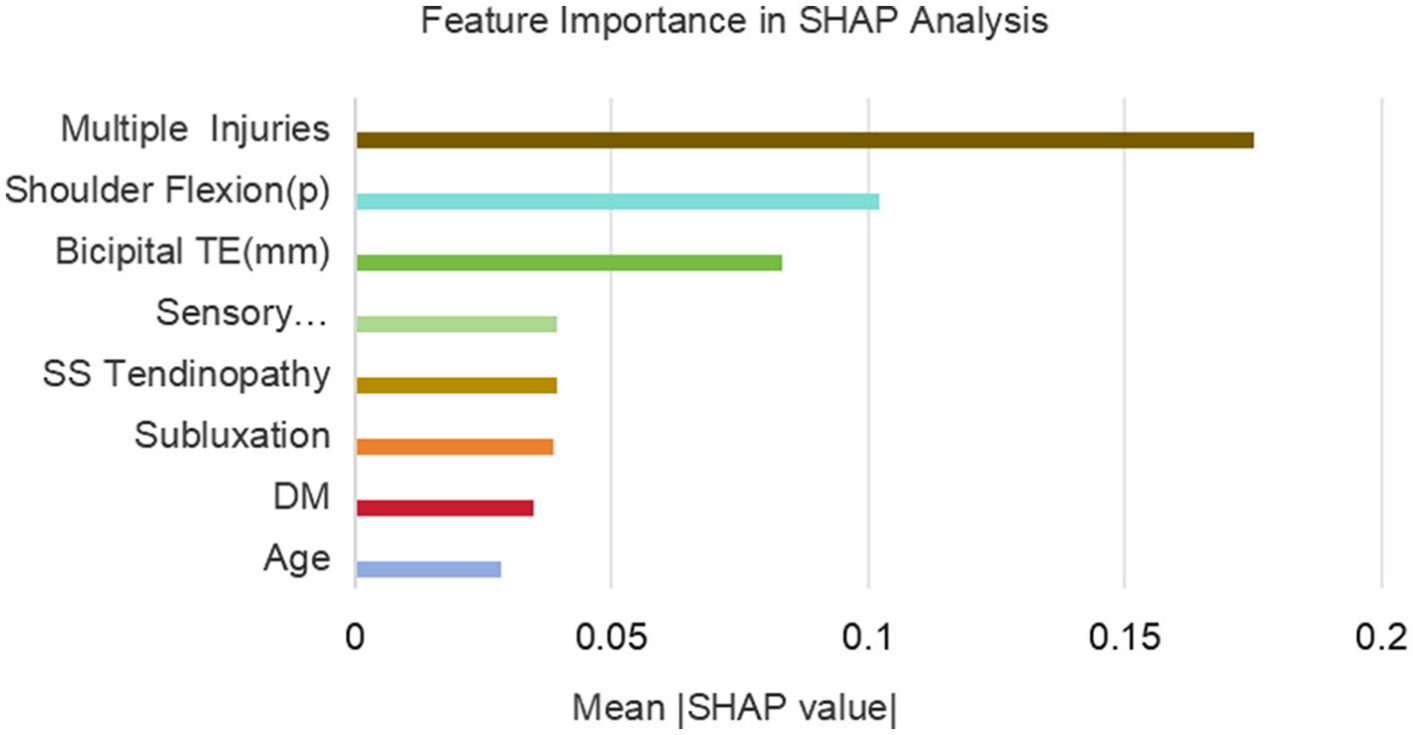
Feature importance in SHAP analysis.

Figure 10 shows the ranking of the importance of the 8 variables included in the RF model. The results indicate that multiple injuries is the most important variable, followed by shoulder flexion (p), bicipital TE, sensory disturbance, SS tendinopathy, subluxation, DM, and age is the least important among all the variables.

## 4 Discussion

This study systematically compared the predictive performance of three machine learning models (LR, SVM, and RF) in the risk stratification of early HSP. Our research results indicated that the machine learning prediction models based on disease feature data and ultrasound imaging results could effectively predict the risk of HSP in stroke patients. Among them, the RF model had the best predictive performance and exhibited good clinical utility and explainability. This is consistent with previous studies, which emphasized that ensemble methods like RF have advantages in handling the inherent complexity, non-linearity, and feature interaction characteristics of clinical data (22, 23).

Given the “black-box effect” in machine learning, enhancing model interpretability is crucial for establishing trust in the model and promoting its reproducibility and practical application (24). In the SHAP analysis of the random forest (RF) model, several key predictive factors were identified, including multiple injuries, shoulder flexion (p), bicipital TE, sensory disturbance, SS tendinopathy, subluxation, and DM. It is important to note that while SHAP analysis effectively quantifies the marginal contribution of each feature to predictive outcomes, there remains a risk of feature weight allocation bias when features are highly correlated or redundant (25). Therefore, we cautiously interpreted these results in clinical context after rationally filtering variables. Upon analysis, the aforementioned factors demonstrate significant clinical relevance supported by existing biomechanical and pathophysiological evidence.

In our study, we observed that 80.7% of hemiplegic patients exhibited abnormal ultrasound images in the early stages of shoulder pathology, a proportion similar to the previously reported range of 60.5%−85% (26, 27), with the number of abnormalities mostly ranging from one to three. Previous studies have revealed that multiple injuries to the shoulder soft tissues detected by ultrasound may play a significant role in the pathogenesis of HSP. For instance, Idowu et al. (28) found through ultrasound examinations of chronic hemiplegic shoulders that most hemiplegic shoulders exhibited more than three abnormal ultrasound images, with some having as many as six. The aforementioned research findings indicate that the affected shoulder of hemiplegic patients already exhibits a certain degree of pathology in the early stages and may sustain further damage during subsequent daily life and rehabilitation processes (29). Therefore, early placement of the healthy limb and strengthening protection of the affected side of the shoulder during daily activities are of vital importance. This can prevent further injuries or aggravation of existing injuries.

Multiple studies have confirmed that limited shoulder joint range of motion (including flexion, abduction, and internal rotation) in the early stages of hemiplegia is closely associated with the occurrence of HSP. Li et al. (7) proposed that early monitoring of shoulder joint range of motion could serve as an effective tool for predicting HSP. We believe that reduced shoulder joint range of motion directly leads to adhesion in the soft tissues surrounding the joint, triggering inflammatory responses and pain (30). Meanwhile, due to the damage of upper motor neurons in hemiplegic patients, the shoulder muscles suffer from either atonic paralysis or spasticity, causing the humeral head to lose its normal support and prone to downward subluxation. This structural change will pull the joint capsule, ligaments and surrounding nerves, such as the axillary nerve, directly triggering pain (31, 32).

Additionally, bicipital tendon effusion and SS Tendinopathy, two common ultrasound findings in hemiplegic shoulder pain, have also been identified as important predictive factors by SHAP analysis. Their high prevalence (3) and strong correlation with pain scores (33) suggest that it may directly contribute to the onset of pain through inflammatory responses and mechanical imbalances.

Post-stroke sensory disturbance are common in hemiplegic patients, including tactile, temperature sensation and proprioception, etc. They may intensify pain perception through central sensitization mechanisms (34). Meanwhile, proprioceptive disorders can also lead to decreased stability of the shoulder joint, increase the risk of soft tissue injury, and subsequently trigger pain (35, 36). Furthermore, DM is an independent risk factor for stroke (37). After stroke, diabetic patients are prone to more severe neurological deficits. This adverse prognosis may indirectly affect the occurrence and development of HSP through mechanisms such as chronic inflammation, accumulation of advanced glycation end products (AGEs), and oxidative stress (38).

From a clinical perspective, the explainability of the RF model, as revealed through SHAP analysis, significantly enhances its practical utility in guiding targeted interventions. For instance, in hemiplegic patients with multiple soft tissue injuries in the shoulder joint identified via ultrasonography, special attention should be paid to avoiding isolated shoulder exercises during rehabilitation. Instead, whole-body coordinated movements, such as balance training and core muscle activation, should be prioritized to mitigate excessive shoulder compensation, thereby reducing the risk of developing HSP.

While the current study has achieved certain progress, its limitations must be carefully considered. Although the sample size of 303 cases generally meets the statistical requirements of the predefined analytical framework, the single-center clinical data collection approach may still constrain the model's external validity. To address this limitation, recent research could prioritize exploring clinically plausible synthetic data generation methods (such as those employing data balancing and generative AI techniques) to produce virtual samples that closely match the distribution of real-world data. This approach would help meet the sample size requirements for achieving predefined statistical power. By enhancing the internal variability coefficient of the dataset without relying on costly multicenter collaborations, this method could improve the model's generalization capability. The efficacy of such approaches has already been validated in numerous machine learning predictive modeling studies (39, 40).

Looking ahead, we hope to continuously refine the predictive model through multicenter external validation, integration of dynamic biomechanical parameters (e.g., electromyography, gait analysis), and longitudinal follow-up evaluations in future research.

In summary, the RF-based predictive model provides a clinically interpretable tool for early risk stratification of HSP, facilitating personalized rehabilitation strategies and resource allocation. By integrating biomechanical, imaging, and clinical data, we hope that this approach will promote proactive management of HSP, ultimately improving patient outcomes and reducing healthcare burdens.

## Data Availability

The datasets presented in this study can be found in online repositories. The names of the repository/repositories and accession number(s) can be found in the article/Supplementary material.

## References

[1] Abdelhakiem NM Mustafa Saleh MS Shabana MMA Abd El Wahaab HA Saleh HM. Effectiveness of a high-intensity laser for improving hemiplegic shoulder dysfunction: a randomized controlled trial. Sci Rep. (2024) 14:7346. 10.1038/s41598-024-57453-9 38538637 PMC10973414

[2] Khatooni M Dehghankar L Samiei Siboni F Bahrami M Shafaei M Panahi R. Association of post-stroke hemiplegic shoulder pain with sleep quality, mood, and quality of life. Health Qual Life Outcomes. (2025) 23:32. 10.1186/s12955-025-02367-x 40188095 PMC11972523

[3] Lin T-Y Shen P-C Chang K-V Wu W-T Özçakar L. Shoulder ultrasound imaging in the post-stroke population: a systematic review and meta-analysis. JRM. (2023) 55:jrm13432. 10.2340/jrm.v55.13432 37615388 PMC10461179

[4] Jia F Zhu X-R Kong L-Y Fan J-C Zhu Z-J Lin L-Z. Stiffness changes in internal rotation muscles of the shoulder and its influence on hemiplegic shoulder pain. Front Neurol. (2023) 14:1195915. 10.3389/fneur.2023.1195915 37332999 PMC10272777

[5] Xie H-M Guo T-T Sun X Ge H-X Chen X-D Zhao K-J. Effectiveness of botulinum toxin A in treatment of hemiplegic shoulder pain: a systematic review and meta-analysis. Arch Phys Med Rehabil. (2021) 102:1775–87. 10.1016/j.apmr.2020.12.010 33454279

[6] De Sire A Moggio L Demeco A Fortunato F Spanò R Aiello V. Efficacy of rehabilitative techniques in reducing hemiplegic shoulder pain in stroke: systematic review and meta-analysis. Ann Phys Rehabil Med. (2022) 65:101602. 10.1016/j.rehab.2021.101602 34757009

[7] Li Y Yang S Cui L Bao Y Gu L Pan H. Prevalence, risk factor and outcome in middle-aged and elderly population affected by hemiplegic shoulder pain: an observational study. Front Neurol. (2023) 13:1041263. 10.3389/fneur.2022.1041263 36712437 PMC9879055

[8] Thakkar HK Liao W Wu C Hsieh Y-W Lee T-H. Predicting clinically significant motor function improvement after contemporary task-oriented interventions using machine learning approaches. J NeuroEngineering Rehabil. (2020) 17:131. 10.1186/s12984-020-00758-3 32993692 PMC7523081

[9] Park S Choi J Kim Y You JSH. Clinical machine learning predicting best stroke rehabilitation responders to exoskeletal robotic gait rehabilitation. NeuroRehabilitation. (2024) 54:619–28. 10.3233/NRE-240070 38943406

[10] Chen Y-W Lin K Li Y Lin C-J. Predicting patient-reported outcome of activities of daily living in stroke rehabilitation: a machine learning study. J NeuroEngineering Rehabil. (2023) 20:25. 10.1186/s12984-023-01151-6 36823626 PMC9948491

[11] Håkansson S Tuci M Bolliger M Curt A Jutzeler CR Brüningk SC. Data-driven prediction of spinal cord injury recovery: an exploration of current status and future perspectives. Exp Neurol. (2024) 380:114913. 10.1016/j.expneurol.2024.114913 39097073

[12] Zhang P Zhou Y Ni H Huang Z Tang C Zhuge Q. Altered functional connectivity of brainstem ARAS nuclei unveils the mechanisms of disorders of consciousness in sTBI: an exploratory study. NeuroImage: Clinical. (2025) 46:103787. 10.1016/j.nicl.2025.103787 40262479 PMC12047610

[13] Wang X Zhong J Lei T Chen D Wang H Zhu L. An artificial neural network prediction model for posttraumatic epilepsy: retrospective cohort study. J Med Internet Res. (2021) 23:e25090. 10.2196/25090 34420931 PMC8414301

[14] Salmanpour MR Shamsaei M Hajianfar G Soltanian-Zadeh H Rahmim A. Longitudinal clustering analysis and prediction of Parkinson's disease progression using radiomics and hybrid machine learning. Quant Imaging Med Surg. (2022) 12:906–19. 10.21037/qims-21-425 35111593 PMC8739095

[15] Xie H-M Zhang X-T Xu L Wang N Wang R Jia Z-S. Magnetic resonance imaging findings in painful hemiplegic shoulder patients with or without subluxation: a retrospective cohort study. Front Neurol. (2022) 13:1032676. 10.3389/fneur.2022.1032676 36457870 PMC9705229

[16] Pirzada RH Ahmad B Qayyum N Choi S. Modeling structure–activity relationships with machine learning to identify GSK3-targeted small molecules as potential COVID-19 therapeutics. Front Endocrinol. (2023) 14:1084327. 10.3389/fendo.2023.1084327 36950681 PMC10025526

[17] Li L Wang S Chen J Wu C Chen Z Ye F. Radiomics diagnosis of microvascular invasion in hepatocellular carcinoma using 3D ultrasound and whole-slide image fusion. Small Methods. (2025) 9:e2401617. 10.1002/smtd.202401617 40200669

[18] Van Calster B Wynants L Verbeek JFM Verbakel JY Christodoulou E Vickers AJ. Reporting and interpreting decision curve analysis: a guide for investigators. Eur Urol. (2018) 74:796–804. 10.1016/j.eururo.2018.08.038 30241973 PMC6261531

[19] Zhang W Wang S Wang Y Sun J Wei H Xue W. Ultrasound-based radiomics nomogram for predicting axillary lymph node metastasis in early-stage breast cancer. Radiol Med. (2024) 129:211–21. 10.1007/s11547-024-01768-0 38280058

[20] Xu C Su X Xu Y Ma S Duan W Mo X. Exploring the associations of serum concentrations of PCBs, PCDDs, and PCDFs with walking speed in the US general population: beyond standard linear models. Environ Res. (2019) 178:108666. 10.1016/j.envres.2019.108666 31472363

[21] Huang L Ruan S Xing Y Feng M. A review of uncertainty quantification in medical image analysis: probabilistic and non-probabilistic methods. Med Image Anal. (2024) 97:103223. 10.1016/j.media.2024.103223 38861770

[22] Su Y Li Y Chen W Yang W Qin J Liu L. Automated machine learning-based model for predicting benign anastomotic strictures in patients with rectal cancer who have received anterior resection. Eur J Surg Oncol. (2023) 49:107113. 10.1016/j.ejso.2023.107113 37857102

[23] Yu Y-D Lee K-S Man Kim J Ryu JH Lee J-G Lee K-W. Artificial intelligence for predicting survival following deceased donor liver transplantation: retrospective multi-center study. Int J Surg. (2022) 105:106838. 10.1016/j.ijsu.2022.106838 36028137

[24] Petch J Di S Nelson W. Opening the black box: the promise and limitations of explainable machine learning in cardiology. Can J Cardiol. (2022) 38:204–13. 10.1016/j.cjca.2021.09.004 34534619

[25] Hu X Zhu M Feng Z Stanković L. Manifold-based Shapley explanations for high dimensional correlated features. Neural Networks. (2024) 180:106634. 10.1016/j.neunet.2024.106634 39191125

[26] Pong Y Wang L Huang Y Leong C Liaw M Chen H. Sonography and physical findings in stroke patients with hemiplegic shoulders: a longitudinal study. J Rehabil Med. (2012) 44:553–7. 10.2340/16501977-0987 22674236

[27] Huang Y Liang P Pong Y Leong C Tseng C. Physical findings and sonography of hemiplegic shoulder in patients after acute stroke during rehabilitation. J Rehabil Med. (2010) 42:21–6. 10.2340/16501977-0488 20111840

[28] Idowu BM Ayoola OO Adetiloye VA Komolafe MA. Sonographic evaluation of structural changes in post-stroke hemiplegic shoulders. Pol J Radiol. (2018) 82:141–8. 10.12659/PJR.899684 28382186 PMC5360429

[29] Huang B Gao F. Analysis of the current status of knowledge, attitudes, and practices among stroke-related healthcare professionals in the treatment of shoulder pain in hemiplegic patients. PeerJ. (2024) 12:e18684. 10.7717/peerj.18684 39703917 PMC11657197

[30] Lawrence RL Richardson LB Bilodeau HL Bonath DJ Dahn DJ Em M-A. Effects of scapular angular deviations on potential for rotator cuff tendon mechanical compression. Orthop J Sports Med. (2024) 12:23259671231219023. 10.1177/23259671231219023 38435717 PMC10906059

[31] Tan B Jia L. Ultrasound-guided BoNT-A (Botulinum Toxin A) injection into the subscapularis for hemiplegic shoulder pain: a randomized, double-blind, placebo-controlled trial. Stroke. (2021) 52:3759–67. 10.1161/STROKEAHA.121.034049 34470492

[32] Chuang L-L Chen Y-L Chen C-C Li Y-C Wong AM-K Hsu A-L. Effect of EMG-triggered neuromuscular electrical stimulation with bilateral arm training on hemiplegic shoulder pain and arm function after stroke: a randomized controlled trial. J NeuroEngineering Rehabil. (2017) 14:122. 10.1186/s12984-017-0332-0 29183339 PMC5706163

[33] Vongviboonchai A Saengsuwan J Sirasaporn P. Ultrasonographic characteristics of the shoulder in patients with shoulder pain: a retrospective study comparing younger and older age groups. J Back Musculoskelet Rehabil. (2025) 38:132–8. 10.1177/10538127241296688 39970458

[34] Haslam BS Butler DS Kim AS Carey LM. Somatosensory impairment and chronic pain following stroke: an observational study. Int J Environ Res Public Health. (2023) 20:906. 10.3390/ijerph20020906 36673661 PMC9859194

[35] Bastos VS Faria CDCM Faria-Fortini I Scianni AA. Prevalence of sensory impairments and its contribution to functional disability in individuals with acute stroke: a cross-sectional study. Rev Neurol. (2025) 181:210–6. 10.1016/j.neurol.2024.12.001 39765442

[36] Arya KN Pandian S Joshi AK Chaudhary N Agarwal G Ahmed SS. Sensory deficits of the paretic and non-paretic upper limbs relate with the motor recovery of the poststroke subjects. Top Stroke Rehabil. (2024) 31:281–92. 10.1080/10749357.2023.2253629 37690032

[37] Mavridis A Viktorisson A Eliasson B Von Euler M Sunnerhagen KS. Risk of ischemic and hemorrhagic stroke in individuals with type 1 and type 2 diabetes: a nationwide cohort study in Sweden. Neurology. (2025) 104:e213480. 10.1212/WNL.0000000000213480 40080734 PMC11907640

[38] Yoshikawa T Mifune Y Inui A Nishimoto H Yamaura K Mukohara S. Influence of diabetes-induced glycation and oxidative stress on the human rotator cuff. Antioxidants. (2022) 11:743. 10.3390/antiox11040743 35453426 PMC9032678

[39] Karlberg B Kirchgaessner R Lee J Peterkort M Beckman L Goecks J. SyntheVAEiser: augmenting traditional machine learning methods with VAE-based gene expression sample generation for improved cancer subtype predictions. Genome Biol. (2024) 25:309. 10.1186/s13059-024-03431-3 39696541 PMC11658131

[40] Trabassi D Castiglia SF Bini F Marinozzi F Ajoudani A Lorenzini M. Optimizing rare disease gait classification through data balancing and generative AI: insights from hereditary cerebellar ataxia. Sensors. (2024) 24:3613. 10.3390/s24113613 38894404 PMC11175240

